# Clonic Spasm of the Muscles of the Eustachian Tube

**Published:** 1871-02

**Authors:** 


### Clonic Spasm of the Muscles of the Eustachian Tube.

D. Politzer, in the *Wien. Med. Presse*, 1870–XX., relates the case of a girl of twelve years who had experienced for five months an almost rhythmical ticking sound in the left ear, that never declined in intensity, and which, during the short periods it ceased, could be voluntarily reproduced. During sleep it was entirely suspended. A hermetically closed manometer tube, containiug a drop of colored fluid, when introduced into the external meatus of the ear, gave no evidence of any movemont of the inclosed drop; it was evident, therefore, that the morbid sound in the ear did not result from any abnormal contraction of the tensor tympani muscle. Neither was the ticking sound dependent upon increased arterial pulsation, as it did not correspond in time with the pulsations at the wrist. The cause of the ticking must hence be sought for within the Eustachian tube. On examination, a convulsive movement of the left half of the arch of the palate was observed to occur isochronously with the abnormal sound in the left ear, the convulsive movement of the palate ceasing with the cessation of the latter, while the latter was stopped, when the velum palati was drawn up and rendered tense during the intonation of the vowels *a*, *e*, *i*, as well as by a voluntary effort of the muscles of the tube. The same has also occurred when, by the finger, the palate was pressed upwards. The case was evidently, therefore, one of clonic spasm of the muscles of the Eustachian tube. It was cured in a short time by Faradaic electricity.—*Centbltt. f. d. Medicinish. Wissenchftn. American Journal ibid.*

